# Role of Tidal Volume on Ventilator-Induced Lung Injury Under Heterogeneous Immunological Capabilities: A Mathematical Model Study

**DOI:** 10.3390/life15060835

**Published:** 2025-05-22

**Authors:** Yao Yu, Yuxi Liu, Fei Lu, Luping Fang, Gangmin Ning, Qing Pan

**Affiliations:** 1 College of Information Engineering, Zhejiang University of Technology, 288 Liuhe Road, Hangzhou 310023, China; 2 Moganshan Institute, Zhejiang University of Technology, 926 Changhong East Road, Deqing 313200, China; 3 Department of Biomedical Engineering, Key Laboratory of Biomedical Engineering of Ministry of Education, Zhejiang University, 38 Zheda Road, Hangzhou 310027, China; 4 Zhejiang Provincial Collaborative Innovation Center for High-End Digital Intelligence Diagnosis and Treatment Equipment, Hangzhou 310023, China

**Keywords:** mechanical ventilation, ventilator-induced lung injury, mathematical model

## Abstract

Mechanical ventilation, a lifesaving intervention for critically ill patients, can also cause ventilator-induced lung injury (VILI), which is related to its interplay with immune system. To explore this relationship, we established a model consisting of two subsystems: a lung immune response system (LIRS), elucidating the interactions between immune cells and epithelial cells within the pulmonary environment, and a tidal volume damage system (TVDS), quantifying alveolar epithelial damage by simulating the detrimental effects of mechanical ventilation across varying tidal volumes. The integrated framework presents a novel lens for examining the interplay between heterogeneous immunological capabilities and tidal volume. The model reveals distinct responses among the individuals with varying immune capacities when subjected to identical tidal volumes, manifesting in diverse outcomes for healthy epithelial cells (*eh*). Analysis of simulation results shows a weak correlation between initial immune cell variables and *eh*, and a stronger correlation between *eh* and baseline repair capacity. Furthermore, while the initial immune state of lung tissue moderately influences epithelial health, the heterogeneous and inherent immunological competence of the tissue plays a pivotal role in shaping the diverse patterns observed in the development and severity of VILI. This insight highlights the importance of tailoring therapeutic strategies to individual immunological profiles, thereby optimizing patient outcomes.

## 1. Introduction

Mechanical ventilation is a lifesaving intervention, enhancing lung function, relieving respiratory distress, and supporting recovery in clinical settings. However, its prolonged or inappropriate use can lead to the development of ventilator-induced lung injury (VILI) [1], a consequence characterized by inflammatory responses and lung tissue damage induced by mechanical ventilation forces. Given the well-established occurrence of VILI across clinical and experimental settings [2], elucidating its pathophysiological mechanisms, especially in the context of acute respiratory distress syndrome (ARDS) with hallmark features of severe inflammation and impaired gas exchange, is imperative. The incidence of VILI varies among different populations [3], highlighting the complexity of this condition. Extensive research endeavors have been undertaken to comprehend the mechanisms underpinning VILI and to investigate prevention and treatment strategies [4,5], with a burgeoning emphasis on the pivotal role of immunological heterogeneity in modulating VILI outcomes. However, extensive research on VILI mechanisms and interventions faces ethical and technological barriers in humans. Animal models, though informative, are costly and time-consuming. Mathematical modeling offers an effective alternative, enabling in silico understanding of VILI pathophysiology, therapeutic predictions, and optimization of ventilation parameters, thereby mitigating VILI risks.

Mathematical analysis and simulations can replicate known biology and predict responses to interventions [6]. In the field of lung pathophysiology, Nieman et al. developed a model to link lung–blood dynamics, providing insights into the interplay of inflammation and acute lung injury [7]. Parallelly in the domain of immune response, Cantone et al. introduced mathematical modeling frameworks that can be used for investigating molecular and cellular mechanisms underlying bacterial lung infection [8]. Furthermore, mathematical modeling has been extensively applied to decipher the dynamic fluctuations of the immune system during various lung infections and injuries [9], with a particular focus on the relationship between VILI and the immune response.

In the realm of VILI modeling, Minucci et al. developed a lung immune response system (LIRS) model that elucidates the interplay between the immune system and the injured lung site, shedding light on the contribution of epithelial injury to VILI pathogenesis [10]. However, their model lacks the link between the development of VILI and the tidal volume of ventilation. Conversely, Bates et al. devised a tidal volume damage system (TVDS) model that incorporates both the initiation and recovery phases of VILI at the cellular level [11]. This model offers a perspective for quantifying the damage inflicted by tidal volumes. Nevertheless, this model overlooked the immune responses that occur at the whole-lung level, which are crucial in understanding and mitigating VILI.

Given the shortcomings identified in the existing researches, our primary contribution lies in establishing a coupling boundary between the LIRS and TVDS models by introducing a ventilation-induced lung injury rate (*sd*) that synchronizes epithelial damage with immune responses in real time. This integrated model is parameterized based on mice experimental data, reinforcing its ability to assess the impact of tidal volume on the severity of VILI. Based on the integrated model, we extend our analysis by simulating the heterogeneous responses of samples with varying immune capacities to identical tidal volumes, thereby elucidating the impact of immunological heterogeneity on VILI outcomes. Expanding upon this foundation, we conduct analysis by simulating the heterogeneous responses of samples, each endowed with distinct immune capacities, to uniform tidal volumes. This approach reveals the impact of immunological heterogeneity on VILI, with the tissue’s inherent immunological competence playing a pivotal role in shaping the diverse patterns observed in both the development and severity of VILI.

## 2. Methods

### 2.1. Model Description

**Figure 1:**
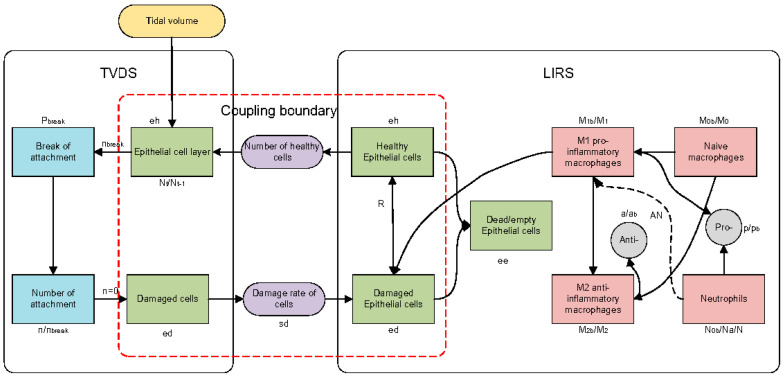
Schematic diagram of model. TVDS: tidal volume damage system, LIRS: lung immune response system. Green squares represent epithelial cells; red squares represent immune cells; gray squares represent anti-inflammatory mediators and pro-inflammatory mediators; blue squares represent the change of intercellular connectors; purple squares represent the quantitative concept of coupling boundary (quantification of healthy epithelial cells and quantification of injury rate); yellow square represents tidal volume. Dotted line signifies the indirect influence of neutrophils on the phenotypic transition of M1 to M2.

The schematic diagram of the proposed model is presented in Figure 1. The model comprises three pivotal modules: the TVDS, which simulates the ventilator-induced damage to alveolar epithelial cells; the LIRS, which captures the interactions between immune cells and lung epithelial cells in the pulmonary environment, and their coupling boundaries highlighted by the red frame in Figure 1. These two subsystems are interconnected through two physical quantities: (1) the quantity of healthy epithelial cells and (2) the rate of cell damage induced by mechanical ventilation. Table 1 presents a list of 25 state variables in the model.

### 2.2. Lung Immune Response System

The LIRS is developed to investigate the immune response mechanisms associated with VILI. The alveolar space is simplified, allowing us to track the temporal dynamics of three distinct subpopulations over time, measured in hours: healthy epithelial cells (eh), damaged epithelial cells (ed), and dead epithelial cells/empty spaces that necessitate replenishment by healthy cells (ee). These subpopulations are modeled as dimensionless quantities, and their combined proportion within the local space is constrained by the equation ee+eh+ed=1, ensuring the conservation of total epithelial cell capacity. In the LIRS module illustrated in Figure 1, eh transitions into ed and ee due to injury, while ed can either further deteriorate into ee or, in the presence of restorative mediators R, undergo repair and revert to eh.

To capture the interplay between the epithelial cells and the immune response elicited by VILI, we formulate a set of ordinary differential equations (ODEs), denoted as Equation (1), which incorporates multiple immune-related components.

(1) $$\left\{\begin{array}{c} \frac{deh}{dt}=G_{eh}+R_{ed}-I_{immu}-sdeh\\ \frac{ded}{dt}=I_{immu}-R_{ed}-P_{M1-ed}-P_{N-ed}-b_{d}ed+sdeh\\ \frac{dee}{dt}=P_{M1-ed}+P_{N-ed}-G_{eh}+b_{d}ed \end{array}\right.$$

The term $G_{eh}$ represents the logistic growth of epithelial cells, which is constrained by available space and modulated by pro-inflammatory mediators [12,13], as described in Equation (2).

(2) $$G_{eh}=\left(b_{p}+k_{ep}p\right)\left(eh+ed\right)ee$$

Equation (3) characterizes the repair process of damaged epithelial cells [12,14], while Equation (4) models the collateral tissue damage mediated by immune cells, particularly macrophages and neutrophils [15].

(3) $$R_{ed}=ed\left(b_{r}+\frac{k_{er}R}{x_{er}+R}\right)$$

(4) $$I_{immu}=eh\left(\frac{k_{mne}{\left(M_{1}+N\right)}^{2}}{x_{mne}^{2}+{\left(M_{1}+N\right)}^{2}}\right)$$

Equations (5) and (6) describe the phagocytic clearance of damaged epithelial cells (ed) by M1 macrophages and activated neutrophils, respectively [15,16]. The term $s_{d}e_{h}$ accounts for ventilator-induced mechanical injury, whereas $b_{d}e_{d}$ denotes the natural decay of injured epithelial cells. A detailed description of all model parameters is provided in the Appendix A.

(5) $$P_{M1-ed}=k_{em1}M_{1}ed\left(\frac{1}{1+{\left(\frac{a}{a_{\infty }}\right)}^{2}}\right)$$

(6) $${P_{N-ed}=k}_{en}Ned$$

While the interplay between the epithelial cells and the immune response forms the crux of the immune response, the present study specifically shines a spotlight on the three pivotal ODEs that govern the dynamics of epithelial cells. Epithelial cells, being the primary interface between the external environment and the immune system, play a pivotal role in coupling boundary. The comprehensive ODE equations, encompassing the main aspects of the immune response, are provided in Supplementary Material S1.

### 2.3. Tidal Volume Damage System

The TVDS focuses on the cellular dimension of mechanical ventilation-induced lung injury, modelling the direct impacts of tidal volume on alveolar epithelial cells, as shown in Figure 1. The TVDS formulates a monolayer consisting of N=10,000 healthy epithelial cells, with each cell anchored to neighboring structures (including other cells and the basement membrane) via n distinct mechanical attachments. When the monolayer is fully confluent, with all attachments intact, n attains its maximum value of $n_{max}=25$.

In the context of the TVDS model, each tidal volume-induced breath is conceptualized as a recruitment/derecruitment (RD) cycle, with each 0.1 h computational time step encompassing 18 primary RD cycles. Each cycle of RD represents a fundamental unit of primary circulation, causing rhythmic alveolar expansion and contraction [17,18]. This process carries the potential for disruption of intercellular attachments [19], which we quantify through the variable n, representing the number of attachments per cell. The disruption can either be a direct consequence of mechanical damage or mediated through mechano-transduction pathways [20,21]. Initially, the healthy epithelial monolayer maintains a relatively intact state characterized by a high value of *n*, signifying abundant attachments per cell. However, with each RD cycle, the cumulative effect of these disruptions manifests in the form of a decrease in *n*, representing the broken of attachments [22].

During each RD, there exists a probability that $P_{break}$ (where $0<P_{break}<1$), if exceeded by a randomly generate threshold for each cell, leads to a decrease in the number of attachments per cell by one. This probability is not static but rather dynamic, increasing with the progression of disruptions as defined by Equations (7) and (8). As the cumulative count of broken attachments, denoted as $n_{broken}=n_{max}-n,$ gradually accumulates, it drives this dynamic adjustment. This mechanism reflects a positive feedback loop that enhances the likelihood of further attachment disruptions, embodying a “rich-get-richer” dynamic observed in ventilator-induced lung injury scenarios [23]. When n reaches zero for a cell, the total healthy cells (*N*) decrements by one, with $N_{t}$ representing the surviving cells at the moment *t*.

(7) $$P_{break}=k(\frac{1}{n_{max}}+\frac{n_{broken}}{n_{max}})$$

(8) $$k=\left\{\begin{array}{c} 0,\;if\;V_{T}<V_{tcrit}\\ \frac{V_{T}-V_{tcrit}}{V_{T}-V_{tcrit}+A},\;if\;V_{T}\geq V_{tcrit} \end{array}\right.\;$$

### 2.4. Coupling Boundary

Central to coupling boundary is the incorporation of a dynamic feedback mechanism between the LIRS and the TVDS modules. This feedback mechanism reflects the interplay between the tidal volume and the immune responses, which jointly contribute to the progression of VILI. The challenge lies in determining the coupling boundary between these two models, particularly in representations of cell damage by VILI and forming effective feedback loops to simulate the real-time interaction dynamics.

LIRS, which defines the damage rate from ventilation as a constant sd, focuses on a static representation of ventilator-induced injury, limiting its capacity to capture the dynamic nature of the interactions between mechanical ventilation, lung tissue, and the immune system. We address this limitation by incorporating dynamic features into sd. We introduce the concept of the damage rate (sd) parameter into the model of TVDS subsystem. Considering that it is uncertain whether the cell has died when it exists independently (n=0), we adopt an extreme yet pragmatic approach, postulating that a cell is considered damaged when the number of intercellular attachments reaches zero. Subsequently, the sd is computed by tracking the variation in the number of damaged cells across these temporal increments, which is quantified by the coefficient C and defined as:

(9) $$sd=C\times \left(\frac{N_{t-1}-N_{t}}{N_{t-1}}\right)$$

where $N_{t-1}$ indicates the total number of cells at the moment t−1. This parameterization allows us to dynamically assess and update the impact of mechanical ventilation on lung epithelial cells, integrating both immune response dynamics and mechanical stressors into a unified framework.

**Figure 2:**
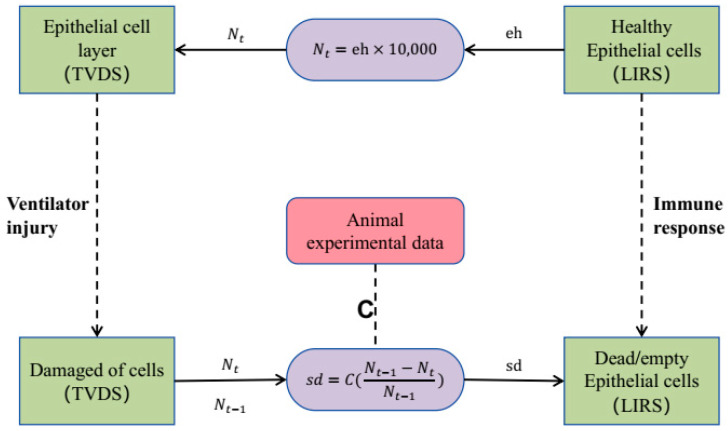
Coupling boundary. Green squares on the left represent epithelial cells within TVDS, while green squares on the right depict epithelial cells within LIRS. Coefficient C of sd is confirmed through animal experimental data.

Forming feedback loops between the immune response model and the tidal volume damage model will enable dynamic, real-time interactions between LIRS and TVDS. At each time step, we integrate update information on healthy epithelial cell (eh) from the LIRS with damage rate (sd) calculations from the TVDS. To harmonize the disparate expression of cell damage between the LIRS and TVDS, we introduce a transformation method that involves multiplying the fractional eh value from LIRS by 10,000 (which means $N_{t}=eh\times 10,000$), thereby deriving the corresponding number of cells updated in TVDS. As Figure 2 illustrates, we continuously update the number of cells undergoing damage from TVDS and eh value from LIRS after each temporal step. This straightforward conversion approach ensures consistency and comparability in quantifying cell damage across these two systems.

### 2.5. Model Parameterization

In the overarching system architecture, the LIRS module incorporates 66 parameters and the TVDS module has three parameters. Furthermore, the coupling boundary module introduces a parameter, denoted as C.

In TVDS, the parameter A, as presented in Equations (7) and (8), was assigned a value of 4.5 mL/kg, which is derived from experimental data using mice weighing approximately 22 g [11]. Additionally, the critical tidal volume ($V_{tcrit}$), as presented in Equation (8), was set at 4 mL/kg, based on the principles of lung-protective ventilation, which advocates tidal volumes ranging from 4 mL/kg to 8 mL/kg [5].

Regarding the selection of the coefficient C of the coupling boundary, we conducted an experimental analysis. Firstly, we undertook a quantitative analysis to determine the number of samples whose end-ventilation hold (eh) scores, within 200 experimental groups, aligned with mice experimental data [24] following two hours of ventilation using distinct tidal volumes. Specifically, we categorized samples into two sets: low tidal volume group, encompassing those with eh scores consistent with the VILI severity scores, which are defined in Table 2, between 1 and 2 distribution after 2 h of ventilation using a tidal volume of 6 mL/kg, and high tidal volume group, comprising samples whose eh scores conform to the VILI severity scores between 2 and 3 distribution subsequent to 2 h of ventilation at 11 mL/kg [24]. Subsequently, we computed the extent of overlap between low tidal volume group and high tidal volume group, defining the coincidence rate as the proportion of overlapping groups relative to the total number of samples in their respective experimental datasets. The average coincidence rate represents the average of the two coincidence rates. Consequently, the coefficient C corresponding to the peak value of the average coincidence rate was selected as the most suitable parameter.

**Figure 3:**
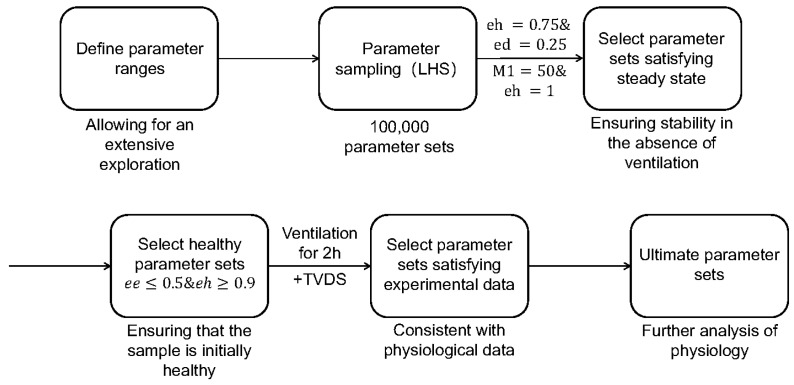
Flowchart of refining parameter sets. Parameter sets of LIRS are originally generated through Latin hypercube sampling (LHS), and they go through two successive screening stages within the LIRS model to identify those parameter sets that represent a healthy and steady-state lung condition before ventilation. Subsequently, the refined parameter sets are further evaluated using the model which coupled LIRS and TVDS, ensuring consistent with experimental data. Then these ultimate parameter sets are used to simulate and analyze.

The LIRS module encapsulates 66 parameters, related to immunological responses, which collectively represent the innate immune competence of a subject. And the parameter sets utilized in this study are drawn from predefined ranges, as detailed in Supplementary Material S2, allowing for an extensive exploration of diverse dynamics and outcomes pertaining to the modeled immune cell populations. These ranges are set to ensure that the resulting sampling encompassed a broad spectrum of behaviors and outcomes [10]. Recognizing that not all parameter combinations mirror physiological realities, we adopted a series of screening methods, as presented in Figure 3, that eliminates parameter sets that fail to adhere to established physiological norms.

Firstly, we implemented Latin hypercube sampling (LHS) using the functions proposed by Marino et al. [6], to generate 100,000 parameter sets within the specified ranges from Supplementary Material S2. These raw parameter sets served as the foundation for our investigations, as depicted in the second module of Figure 3. Our simulations aimed to explore how baseline lung health influences both ventilation response and post-ventilation recovery dynamics.

To ensure stability in the absence of ventilation, we conducted a simulation of the LIRS model across 100,000 parameter sets, excluding mechanical ventilation. A criterion for numerical steady state was established, requiring that the $l^{2}$-norm of the difference between each mesh point in the last 100 h of the simulation and the last point (hour 800) is less than 0.1 [10]. This was established based on minimal fluctuations in all variables during the final 100 h of the simulation, ensuring stability in the absence of ventilation. Additionally, we introduced two distinct initial conditions to further validate stability. The first condition, all variables set to zero except for eh=0.75 and ed=0.25, involved an insult to epithelial cells without an activated immune response. The second condition, all variables set to zero except for M1=50 and eh=1, simulated an activated immune response alongside healthy tissue [10]. If our set satisfied steady conditions with all these initial conditions, then the parameter set was accepted, and the relevant initial variables were set to the variable values at 800 h [10].

To ensure biological plausibility and maintain focus on scenarios where the tissue remained largely healthy, parameter sets resulting in an initial variable with ee (epithelial empty/dead cells) exceeded 50% were excluded from further analysis [10]. Then, the selected parameter sets were matched to their respective initial variable values and simulated for another 200 h without ventilation. This allowed for the identification of parameter sets capable of maintaining epithelial cell health (eh≥90%), ensuring samples with the initial health status.

To correspond to the adopted mice experimental data [24], the integrated model (LIRS+TVDS) underwent simulations at two distinct tidal volumes, namely 6 mL/kg and 11 mL/kg, over a period of two hours. Following this, the simulation outcomes were categorized into four distinct groups based on the eh value recorded at the two-hour mark, as tabulated in Table 2. Within these categorizations, specific parameter sets were identified that exhibited concordance with the lung epithelial cell injury scores observed under both ventilation conditions of 6 mL/kg and 11 mL/kg. These parameter sets mirror the VILI severity scores falling within the range of 1 to 2 when ventilated at 6 mL/kg and corresponded to scores between 2 and 3 when ventilated at 11 mL/kg [24], thereby demonstrating substantial agreement with the mice experimental data.

## 3. Results

### 3.1. Validity of the Coupling Boundary

**Figure 4:**
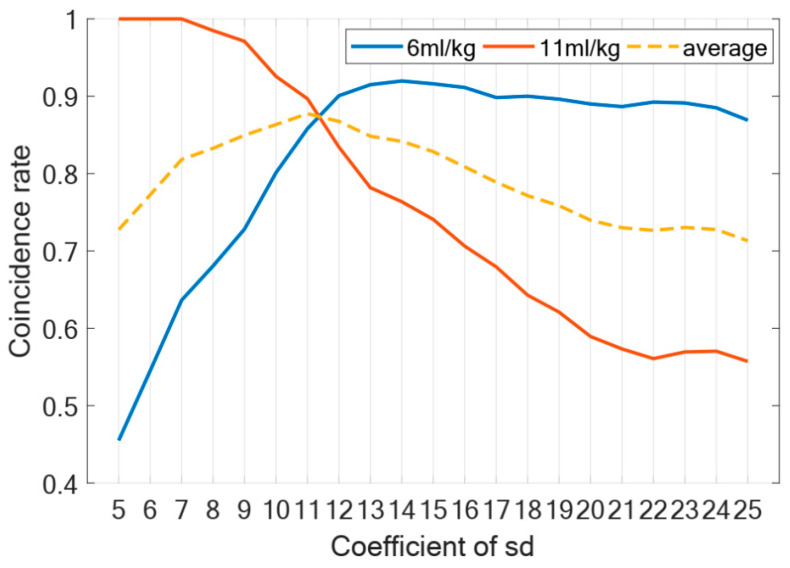
Coefficient of sd. Figure shows change trend of coincidence rate under different coefficients of sd. Blue line represents change curve of data coincidence rate at tidal volume of 6 mL/kg when ventilated for two hours; red line represents tidal volume of 11 mL/kg; dotted line represents average coincidence rate.

To validate the coupling boundary in the model, we conducted a comprehensive analysis of the coefficient C. Firstly, regarding the selection of the coefficient C, Figure 4 presents the change trend of the coincidence rate. The coincidence rate, defined as the proportion of samples (out of 200 test cases) that showed matching eh score distributions between our model’s 2 h mechanical ventilation simulation and Santos’ experimental data after ventilation, was used to determine the optimal C value. According to Figure 4, the coefficient C corresponding to the peak value of the average coincidence rate is 11, so we choose C=11 as the most suitable coefficient.

**Figure 5:**
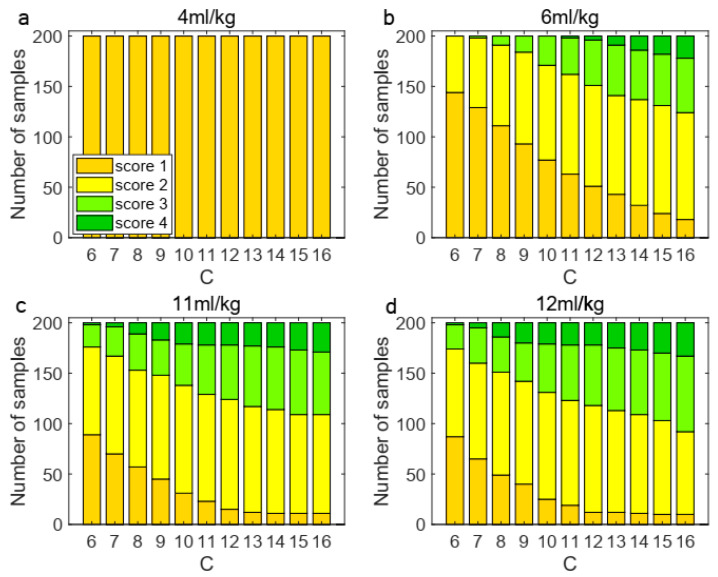
Sensitivity analysis of sd coefficient under four tidal volumes. The classification of 200 samples changed under different sd coefficients. (**a**) At 4 mL/kg, the classification remained unchanged; (**b**) under 6 mL/kg, the distribution shifted; (**c**) When it was increased to 11 mL/kg, the classification changed significantly; (**d**) at 12 mL/kg, same to 11 mL/kg.

Secondly, we performed a sensitivity analysis under four tidal volumes by testing C values ranging from 6 to 16, with 11 as the midpoint. Figure 5 presents a sensitivity analysis of the impact of varying sd coefficients on sample classification. The results, as depicted in Figure 5, reveal two key findings: (1) at 4 mL/kg tidal volume (not higher than $V_{tcrit}$ which represents TVDS’s injury threshold), all samples maintained score 1 regardless of C variation, confirming our threshold design; and (2) above-threshold ventilation showed progressive score increases with rising C values, which demonstrates a significant sensitivity in sample classification to alterations in the sd coefficient. The results demonstrated significant sensitivity of sample classification to variations in the sd coefficient, confirming that this parameter plays a crucial role in determining the classification outcomes of the process.

**Figure 6:**
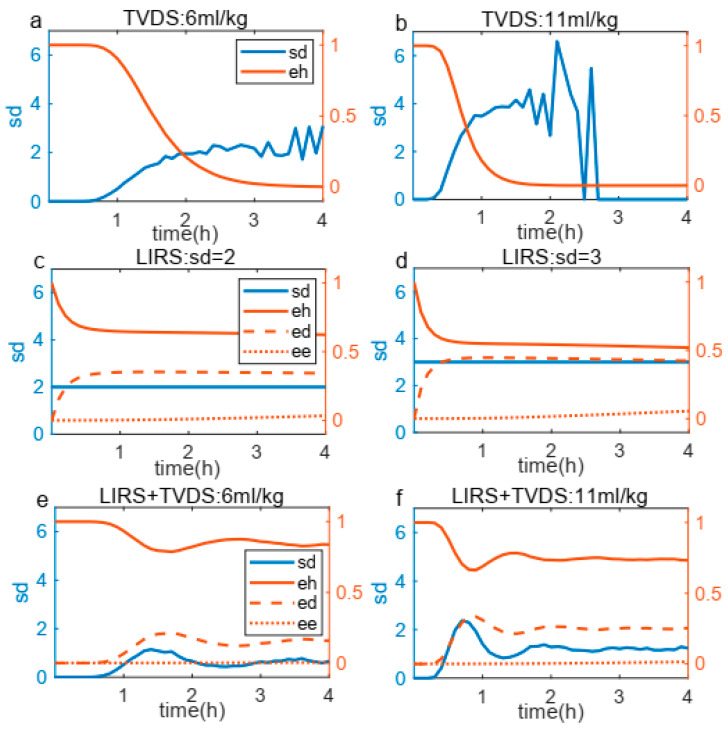
Relationship between sd and eh, ed, ee during 4 h mechanical ventilation. (**a**,**b**) TVDS system response under two tidal volumes, showing earlier *eh* depletion and more pronounced sd fluctuations at higher tidal volume. (**c**,**d**) LIRS system behavior under different sd conditions, revealing time-course of eh, ed, and ee. (**e**,**f**) Coupled system (LIRS+TVDS) results that integrate features from both subsystems, ultimately reaching stabilization during 4 h ventilation.

To validate the real-time interactive capabilities of the coupling boundary, we analyzed a representative sample’s temporal evolution of key parameters (sd, eh, ed, and ee) during 4 h of ventilation across three configurations: (a–b) TVDS-only, (c–d) LIRS-only, and (e–f) the coupled LIRS+TVDS system (Figure 6). In the TVDS-only system (Figure 6a,b), higher tidal volumes accelerate eh depletion while amplifying sd fluctuations. During late-phase ventilation, the system exhibited particularly dramatic sd oscillations because changes in the critically diminished eh values triggered sd instability. This unstable progressively intensified until the depletion of eh to zero. The LIRS-only system (Figure 6c,d), maintaining a stable inverse correlation between eh and ed regardless of sd levels, with ee remaining remarkably constant throughout ventilation. The coupled LIRS+TVDS system (Figure 6e,f) exhibited a composite behavior incorporating features from both subsystems: it preserved the TVDS-derived ventilation-volume-dependent sd fluctuations while maintaining the LIRS-type inverse eh-ed relationship with stable ee. This confirms successful integration of both systems’ dynamics through the coupling boundary. Interestingly, during the late phase of prolonged ventilation, the coupled system exhibited a relatively stabilized state, demonstrating a dynamic equilibrium between ventilator-induced lung injury and immune-mediated repair capacity.

### 3.2. Effect of Immunological Capacity on the Degree of VILI

**Figure 7:**
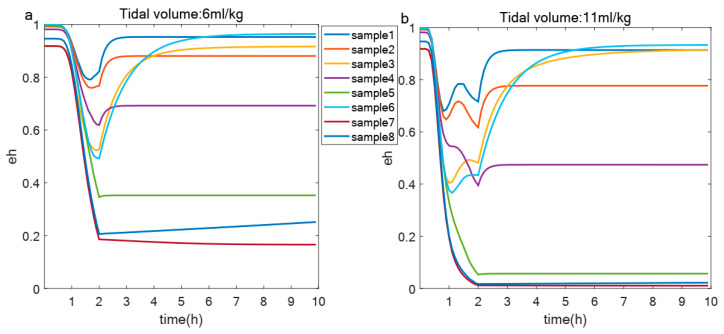
Changes of eh in different samples under same tidal volume. (**a**,**b**) Evolution of eh over 10 h in healthy epithelial cells from eight samples under two different tidal volumes: 6 mL/kg (**a**) and 11 mL/kg (**b**).

After applying the screening criteria outlined in Section 2.5, parameter sets were identified that, when subjected to a two-hour ventilation period followed by a recovery phase, exhibited varying levels of eh. Each parameter set represents an individual with unique autoimmune capabilities and initial immune states. To illustrate the impact of these differing immunological profiles on eh outcomes, as depicted in Figure 7, we randomly selected eight samples to present their distinct eh responses under two tidal volumes: 6 mL/kg and 11 mL/kg. This figure shows that variations in immune competence and initial immune status can lead to different eh trajectories among individuals.

**Figure 8:**
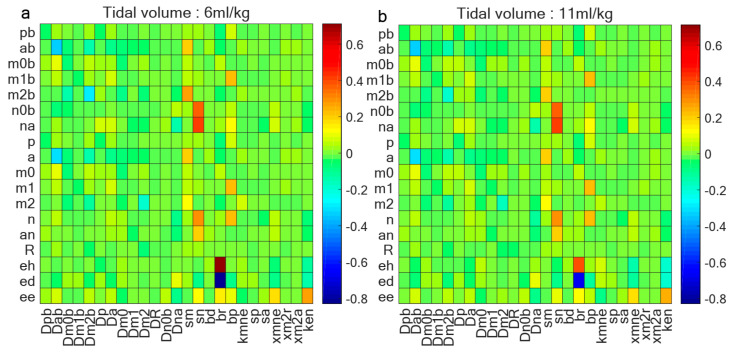
Correlation coefficient between 18 variables and parameters. (**a**) Correlation coefficient between variables and some parameters when tidal volume is 6 mL/kg and ventilation is 2 h; (**b**) correlation coefficient between variables and some parameters when tidal volume is 11 mL/kg and ventilation is 2 h. Baseline repair capacity (br) of damaged cells stands out as determinant of lung epithelial cell health; this correlation diminishes as tidal volume increases.

To gain insights into the influence of autoimmune capacity on eh, we conducted some analyses encompassing various parameters and variables. Figure 8 explores the correlations among these 18 variables and some parameters, with Figure 8a focusing on the data acquired at 6 mL/kg and Figure 8b at 11 mL/kg. The detailed parameter descriptions in Figure 8 are provided in the Appendix A. The analysis reveals that the baseline repair capacity (br) of damaged cells emerges as the most significant determinant of lung epithelial cell health. Under protective ventilation (6 mL/kg), cellular repair mechanisms dominate in maintaining epithelial homeostasis, as evidenced by strong positive correlations between br and epithelial health markers. However, this protective correlation progressively weakens at higher tidal volumes (11 mL/kg), indicating that excessive mechanical strain eventually overwhelms the repair capacity.

## 4. Discussion

The present study has established a mathematical framework that dynamically simulates the immune response of lung tissue to varying tidal volumes during mechanical ventilation. This model provides unique insights into the interplay between the immune system and lung injury, illuminating potential pathways for the development of targeted therapeutic strategies aimed at mitigating the deleterious effects of mechanical ventilation. As demonstrated in Figure 6a,b, higher tidal volumes accelerate the transition from a steady state to an exponential decline in eh, underscoring the exacerbating effects of mechanical stress on VILI [18,24]. Intriguingly, subsequent to this decline, eh exhibits a resurgence, which underscores the inherent repair capabilities of the sample. This phenomenon is further corroborated in Figure 6e,f, where under prolonged ventilation, eh plateaus following the turmoil, showcasing the system’s inherent recovery mechanisms [12,14].

The model also uncovers important heterogeneity in immunological responses, as Figure 7 provides insight into the heterogeneous immunological capabilities across diverse samples, evidenced by disparities in eh under standardized tidal volumes. Furthermore, Figure 8 underscores the importance of innate immune repair mechanisms, as baseline repair capacity emerges as the strongest correlate of epithelial health [12,25]. Notably, this correlation diminishes with increasing tidal volume, underscoring the relationship between ventilation parameters and the effectiveness of innate repair processes. The transition from repair-dominated to injury-dominated responses reveals that ventilator-induced damage surpasses the compensatory ability of cellular repair systems.

Our findings emphasizing the importance of the inherent immunological competence to respond to injury. This highlights the role of personalized therapeutic strategies that account for individual immunological profiles, as they have the potential to enhance treatment efficacy, mitigate VILI, and safeguard lung health. Our observations indicate that despite similar ventilator settings, patients may display vastly different immune responses, revealing the intricate and multifaceted nature of the immune system’s response to lung injury. Future investigations into the genetic, environmental, and epigenetic factors that contribute to this heterogeneity could pave the way for precision medicine approaches, tailored to address the unique immunological needs of critically ill patients requiring mechanical ventilation.

Despite our current model in dynamically simulating the immune response of lung tissue to varying tidal volumes during mechanical ventilation, it is crucial to acknowledge its limitations. One primary limitation revolves around the simplification of ventilator settings, focusing solely on a single parameter, whereas clinical practice often involves intricate protocols incorporating multiple adjustable factors such as tidal volume, plateau pressure, driving pressure, and positive end-expiratory pressure, each influencing the severity of VILI [5,26]. Future development of our model, enriched with these additional ventilator parameters, would undoubtedly yield a more realistic representation of the clinical scenario, deepening our comprehension of the interplay between ventilator settings and immune responses. Furthermore, by investigating the applicability of our model to pulmonary diseases beyond VILI, such as neuromuscular respiratory disease and chronic obstructive pulmonary disease (COPD), with diverse experimental data, we aim to uncover insights between ventilator settings and the immunological profiles and pathophysiological mechanisms of these diseases.

## 5. Conclusions

The present study represents an advancement to create a novel mathematical model that dynamically simulates the immune response of lung tissue to varying tidal volumes during mechanical ventilation. This model enhances our understanding of the dynamic interplay between the immune system and mechanical ventilation in the context of ventilator-induced lung injury. By simulating the heterogeneous immune responses of samples with diverse immunological capacities to identical tidal volumes, we illuminate the pivotal role of immunological heterogeneity in shaping VILI outcomes. While recognizing the limitations of the current work, our findings lay a foundation for future research endeavors aimed at deciphering the complexities of immune responses to lung injury and guiding the development of more targeted and effective therapeutic interventions. The integration of additional ventilator parameters, and a deeper exploration of immune heterogeneity, holds immense potential to propel the field forward to improve patients’ outcomes.

## Figures and Tables

**Table 1:** State variables for the model. TVDS (tidal volume damage system) and LIRS (lung immune response system).

TVDS	LIRS	Description
	p_b_	Pro-inflammatory mediators in bloodstream
	a_b_	Anti-inflammatory mediators in bloodstream
	M0_b_	Naive macrophages in bloodstream
	M1_b_	M1 pro-inflammatory macrophages in bloodstream
	M2_b_	M2 anti-inflammatory macrophages in bloodstream
	N_0b_	Unactivated neutrophils in bloodstream
	N_a_	Activated neutrophils in bloodstream
	p	Pro-inflammatory mediators in lung
	a	Anti-inflammatory mediators in lung
	M0	Naive macrophages in lung
	M1	M1 pro-inflammatory macrophages in lung
	M2	M2 anti-inflammatory macrophages in lung
	N	Neutrophils in lung
	AN	Apoptotic neutrophils in lung
	R	Repair mediators in lung
eh	eh	Healthy epithelial cells
ed	ed	Damaged epithelial cells
	ee	Dead epithelial cells/empty space
p_break_		Probability of intercellular attachments breakage
n_break_		Number of broken intercellular attachments
n		Number of intercellular attachments
k		Conform p_break_ to a nonlinear expression constrained to lie between 0 and 1
N_t_		The total number of epithelial cells at the moment *t*
N_t−1_		The total number of epithelial cells at the moment t−1
sd	sd	Damage rate from ventilator

**Table 2:** Definition of four degrees of VILI severity based on eh values at 2 h.

VILI Severity Score	*eh* Values at 2 h
Score 1	$eh\left(2h\right)\geq 75\%$
Score 2	$50\%\leq eh\left(2h\right)<75\%$
Score 3	$25\%\leq \;eh\left(2h\right)<50\%$
Score 4	$eh\left(2h\right)>25\%$

## Data Availability

The raw data supporting the conclusions of this article will be made available by the authors on request.

## Supplementary Materials

The following supporting information can be downloaded at: https://www.mdpi.com/article/10.3390/life15060835/s1, S1: The complete ODE equations of the Lung Immune Response System (LIRS); S2: Parameters in LIRS; S3: Model Validation.

## Appendix A

**Table A1:** Parameters in LIRS that are mentioned in this paper.

Name	Description
$a_{\infty }$	Relative effectiveness of a at inhibiting M0 differentiation to M1
$b_{d}$	Baseline decay of damaged cells
$b_{p}$	Baseline self-resolving repair of epithelial cells
$b_{r}$	Baseline repair of damaged cells
$D_{pb}$	Decay rate of pro-inflammatory mediators in bloodstream
$D_{ab}$	Decay rate of anti-inflammatory mediators in bloodstream
$D_{m0b}$	Decay rate of naive macrophages in bloodstream
$D_{m1b}$	Decay rate of M1 in bloodstream
$D_{m2b}$	Decay rate of M2 in bloodstream
$D_{p}$	Decay rate of pro-inflammatory mediators in lung
$D_{a}$	Decay rate of anti-inflammatory mediators in lung
$D_{m0}$	Decay rate of naive macrophages in lung
$D_{m1}$	Decay rate of M1 in lung
$D_{m2}$	Decay rate of M2 in lung
$D_{R}$	Decay rate of repair mediators in lung
$D_{n0b}$	Decay rate of unactivated neutrophils in bloodstream
$D_{na}$	Decay rate of activated neutrophils in bloodstream
$s_{m}$	Source rate of naive macrophages in bloodstream
$s_{n}$	Source rate of unactivated neutrophils in bloodstream
$k_{ep}$	Rate of self-resolving repair mediated by p
$k_{er}$	Rate of repair of damaged cells by R
$k_{em1}$	Rate of phagocytosis of damaged cells by M1
$k_{mne}$	Rate of collateral damage to epithelial cells by macrophages and neutrophils
$k_{en}$	Rate of phagocytosis of damaged cells by Neutrophils in lung
$s_{p}$	Source rate of background pro-inflammatory mediators in bloodstream
$s_{a}$	Source rate of background anti-inflammatory mediators in bloodstream
$x_{mne}$	Regulates effectiveness of macrophages and neutrophils to damage epithelial cells
$x_{m2r}$	Regulates effectiveness of M2 in bloodstream recruitment by R
$x_{m2a}$	Regulates effectiveness of M2 in bloodstream recruitment by a
$x_{er}$	Regulates effectiveness of repair of damaged cells by R
