# Supplementary material for: Role of Tidal Volume on Ventilator-Induced Lung Injury Under Heterogeneous Immunological Capabilities: A Mathematical Model Study
Source: Life (Basel). 2025 May 22;15(6):835. doi: 10.3390/life15060835 (PMC12194398; doi:10.3390/life15060835)
Supplement: Supplementary file 1 [file life-15-00835-s001.zip › life-3575338-supplementary.pdf]

Supplementary Material

Role of tidal volume on ventilator-induced lung injury under heterogeneous immunological capabilities: a mathematical model study

Yao Yu[1], Yuxi Liu[1,2], Fei Lu[1], Luping Fang[1], Gangmin Ning[3], Qing Pan[1,4*]

1. College of Information Engineering, Zhejiang University of Technology, 288 Liuhe Road, Hangzhou, China

2. Moganshan Institute, Zhejiang University of Technology, 926 Changhong East Road, Deqing, China

3. Department of Biomedical Engineering, Key Laboratory of Biomedical Engineering of Ministry of Education, Zhejiang University, 38 Zheda Road, Hangzhou, China

4. Zhejiang Provincial Collaborative Innovation Center for High-end Digital Intelligence Diagnosis and Treatment Equipment, Hangzhou, China

Corresponding author:

Prof. Qing Pan

College of Information Engineering, Zhejiang University of Technology, 288 Liuhe Road, Hangzhou, 310023, China

Email: pqpq@zjut.edu.cn; Phone: +86-571-85290595

Table of Contents

S1. The complete ODE equations of the Lung Immune Response System (LIRS) 3
1.1. Modeling of lung epithelial cells.....3
1.2. Modeling of macrophages.....4
1.3. Modeling of neutrophils.....5
1.4. Modeling of cellular media.....6
S2. Parameters in LIRS.....8
S3. Model Validation......10

S1. The complete ODE equations of the Lung Immune Response System (LIRS)

1.1. Modeling of lung epithelial cells

In the process of modeling lung epithelial cells, Eq. (1) describes the logical growth process of lung epithelial cells, which is specifically manifested in the diffusion and proliferation of cells to fill the blank space.

$$G_{eh} = \left(b_p + k_{ep}p\right)\left(eh + ed\right)ee \tag{1}$$

Eq. (2) describes the repair process of damaged epithelial cells to healthy epithelial cells. This repair process mainly depends on the inherent self-repair ability of lung epithelial cells.

$$R_{ed} = ed\left(b_r + \frac{k_{er}R}{x_{er}R}\right) \tag{2}$$

Eq. (3) describes the collateral damage to lung epithelial cells caused by immune activities.

$$I_{immu} = eh\left(\frac{k_{mne}\left(M1+N\right)^2}{x_{mne}^2 + \left(M1+N\right)^2}\right) \tag{3}$$

Eq. (4) and eq. (5) respectively represent the phagocytosis of damaged cells by M1 macrophages and activated neutrophils.

$$P_{M1-ed} = k_{em1}M1ed\left(\frac{1}{1+\left(\frac{a}{a_\infty}\right)^2}\right) \tag{4}$$

$$P_{N-ed} = k_{en}Ned \tag{5}$$

The equations (6), (7) and (8) represent the dynamic changes of healthy epithelial cells, damaged epithelial cells and blank space, respectively. These differential equations constitute the core mathematical framework of lung epithelial cell population dynamics.

$$\frac{deh}{dt} = G_{eh} + R_{ed} - I_{immu} - sdeh \tag{6}$$

$$\frac{ded}{dt} = I_{immu} - R_{ed} - P_{M1-ed} - P_{N-ed} - b_d ed + sdeh \tag{7}$$

$$\frac{dee}{dt} = P_{M1-ed} + P_{N-ed} - G_{eh} + b_d ed \tag{8}$$

1.2. Modeling of macrophages

Macrophages exist in the lungs and blood and are important participants in immune response. In the model, the dynamic behavior of macrophages is described by diffusion terms, as shown in equations (9), (10) and (11).

$$S_{M0} = (M0 - M0_b)\left(d_{m0} + \frac{k_{m0pd}p_b}{x_{m0pd} + p_b} + \frac{k_{m0ad}a_b}{x_{m0ad} + a_b}\right) \tag{9}$$

$$S_{M1} = (M1 - M1_b)\left(d_{m1} + \frac{k_{m1p}p_b}{x_{m1p} + p_b}\right) \tag{10}$$

$$S_{M2} = (M2 - M2_b)\left(d_{m2} + \frac{k_{m2r}R}{x_{m2r} + R} + \frac{k_{m2a}a}{x_{m2a} + a}\right) \tag{11}$$

Eq. (12) indicates that M0 macrophages receive pro-inflammatory mediators (PIM) and differentiate into M1 phenotype.

$$\begin{cases} DF_{M1b} = \left(\frac{k_{m0pb}p_b^2}{x_{m0pb}^2 + p_b^2}\right)\left(\frac{1}{1+\left(\frac{a_b}{a_{b\infty}}\right)^2}\right) \\ DF_{M1} = \left(\frac{k_{m0p}p^2}{x_{m0p}^2 + p^2}\right)\left(\frac{1}{1+\left(\frac{a}{a_\infty}\right)^2}\right) \end{cases} \tag{12}$$

Eq. (13) indicates that M0 macrophages receive anti-inflammatory mediators (AIM) and differentiate into M2 phenotype.

$$\begin{cases} DF_{M2b} = \frac{k_{m0ab}a_b^2}{x_{m0ab}^2 + a_b^2} \\ DF_{M2} = \frac{k_{m0a}a^2}{x_{m0a}^2 + a^2} \end{cases} \tag{13}$$

After phagocytosis of apoptotic neutrophils, M1-type macrophages may undergo phenotypic transformation and turn into M2-type macrophages. This dynamic process is simulated by Equation (14).

$$T_{M1-M2} = k_{man}(k_{anm1}ANM1)\left(\frac{1}{1+\left(\frac{a}{a_\infty}\right)^2}\right) \tag{14}$$

Eq. (15) and (16) describe the dynamic behavior of undifferentiated macrophages (M0 type)

in blood and lungs, equations (17) and (18) describe the changes of M1 type macrophages in blood and lungs, and equations (19) and (20) represent M2 type macrophages in blood and lungs.

$$\frac{dM0_b}{dt} = S_{M0} - M0_b\left(DF_{M1b} + DF_{M2b}\right) + s_m - D_{m0b}M0_b \tag{15}$$

$$\frac{dM0}{dt} = -S_{M0} - M0\left(DF_{M1} + DF_{M2}\right) - D_{m0}M0 \tag{16}$$

$$\frac{dM1_b}{dt} = S_{M1} + M0_b DF_{M1b} - D_{m1b}M1_b \tag{17}$$

$$\frac{dM1}{dt} = -S_{M1} + M0DF_{M1} - T_{M1-M2} - D_{m1}M1 \tag{18}$$

$$\frac{dM2_b}{dt} = S_{M2} + M0_b DF_{M2b} - D_{m2b}M2_b \tag{19}$$

$$\frac{dM2}{dt} = -S_{M2} + M0DF_{M2} - T_{M1-M2} - D_{m2}M2 \tag{20}$$

1.3. Modeling of neutrophils

Eq. (21) describes the activation process of neutrophils.

$$A_{N0} = N0_b\left(\frac{k_{n0p}p_b^2}{x_{n0p}^2 + p_b^2}\right)\left(\frac{1}{1+\left(\frac{a_b}{a_{b\infty}}\right)^2}\right) \tag{21}$$

Equations (22) and (23) describe the dynamic behavior of inactive and activated neutrophils.

$$\frac{dN0_b}{dt} = s_n - A_{N0} - D_{n0b}N0_b \tag{22}$$

$$\frac{dNa}{dt} = A_{N0} - k_n Na - D_{na}Na \tag{23}$$

Eq. (24) shows that M1 macrophages phagocytize apoptotic neutrophils, and Eq. (25) shows that M2 macrophages phagocytize apoptotic neutrophils.

$$P_{M1-AN} = k_{anm1} ANM1 \left(\frac{1}{1 + \left(\frac{a}{a_{\infty}} \right)^2} \right) \quad (24)$$

$$P_{M2-AN} = k_{anm2} ANM2 \quad (25)$$

Equations (26) and (27) describe the dynamic behavior of pulmonary neutrophils and apoptotic neutrophils.

$$\frac{dN}{dt} = k_n N_b - k_{an} N - D_n N \quad (26)$$

$$\frac{dAN}{dt} = k_{an} N - P_{M1-AN} - P_{M2-AN} \quad (27)$$

1.4. Modeling of cellular media

The movement of pro-inflammatory mediators between lung chamber and blood chamber is modeled by passive diffusion mechanism, as shown in equations (28) and (29).

$$S_P = d_p \left(p - p_b \right) \quad (28)$$

$$S_A = d_a \left(a - a_b \right) \quad (29)$$

M1 macrophages produce PIM, which up-regulates the activation and migration of macrophages to the injured site, as shown in Eq. (30).

$$\begin{cases} PD_{M1-pb} = k_{pm1} M_{1b} \left(\frac{1}{1 + \left(\frac{a_b}{a_{b\infty}} \right)^2} \right) \\ PD_{M1-p} = k_{pm1} M_1 \left(\frac{1}{1 + \left(\frac{a}{a_{\infty}} \right)^2} \right) \end{cases} \quad (30)$$

Equations (31) and (32) describe the dynamic changes of PIM in blood and lungs, respectively.

$$\frac{dp_b}{dt} = S_P + PD_{M1-pb} + k_{pn} Na + s_p - D_{pb} p_b \quad (31)$$

$$\frac{dp}{dt} = -S_P + PD_{M1-p} + k_{pn}N + k_{pe}ed - D_p p \tag{32}$$

Equations (33) and (34) respectively represent the process of AIM production by M1-type macrophages and M2-type macrophages, and the equations include the reactions of blood and lung chambers.

$$\begin{cases} PD_{M1-ab} = k_{am1}M1_b \\ PD_{M1-a} = k_{am1}M1 \end{cases} \tag{33}$$

$$\begin{cases} PD_{M2-ab} = k_{am2}M2_b \\ PD_{M2-a} = k_{am2}M2 \end{cases} \tag{34}$$

Equations (35) and (36) describe the dynamic changes of AIM in blood and lungs, respectively.

$$\frac{da_b}{dt} = S_A + PD_{M1-ab} + PD_{M2-ab} + s_a - D_{ab}a_b \tag{35}$$

$$\frac{da}{dt} = -S_A + PD_{M1-a} + PD_{M2-a} - D_a a \tag{36}$$

Eq. (36) simulates the process of epithelial cells repair.

$$\frac{dR}{dt} = k_{rm2}M2 - D_R R \tag{37}$$

S2. Parameters in LIRS

Table S1. Parameters in LIRS with short descriptions and ranges.

Name	Description	Range used
$a_{b\infty}$	Relative effectiveness of a_b at inhibiting M_{0b} differentiation to M_{1b}	[0.29,67.35]
a_{∞}	Relative effectiveness of a at inhibiting M_0 differentiation to M_1	[0.13,72.08]
b_d	Baseline decay of damaged cells	$[1.06 \times 10^{-5}, 0.07]$
b_p	Baseline self-resolving repair of epithelial cells	[0,6.20]
b_r	Baseline repair of damaged cells	$[9.79\times 10^{-3}, 4.47]$
d_a	Rate of diffusion for a	[0.19,177.98]
d_p	Rate of diffusion for p	$[0.34, 2.3\times 10^{3}]$
d_{m0}	Rate of diffusion for M_0	[0.24,275.55]
d_{m1}	Rate of diffusion for M_1	$[2.75\times 10^{-3}, 19.8]$
d_{m2}	Rate of diffusion for M_2	[0.14,143.36]
k_{am1}	Production rate of a by M_{1b} & M_1	[0.01,18.01]
k_{am2}	Production rate of a by M_{2b} & M_2	$[2.43\times 10^{-3}, 1.67]$
k_{an}	Rate at which neutrophils become apoptotic	[0.01,50.04]
k_{anm1}	Rate of M1 phagocytosis of AN	$[1.32\times 10^{-3}, 0.69]$
k_{anm2}	Rate of M2 phagocytosis of AN	$[2.71\times 10^{-3}, 7.36]$
k_{em1}	Rate of phagocytosis of damaged cells by M1	[0.01,16.03]
k_{en}	Rate of phagocytosis of damaged cells by N	[0.01,16.03]
k_{ep}	Rate of self-resolving repair mediated by p	[0,4.30]
k_{er}	Rate of repair of damaged cells by R	$[1.47\times 10^{-3}, 1.08]$
x_{er}	Regulates effectiveness of repair of damaged cells by R (Hill-type constant)	$[7.23\times 10^{-3}, 4.13]$
k_{m0a}	Rate of differentiation of M0 by a	[0.01,89.07]
x_{m0a}	Regulates effectiveness of differentiation of M0 by a (Hill-type constant)	[0.16,136.83]
k_{m0ab}	Rate of differentiation of M0b by ab	[1.15,436.59]
x_{m0ab}	Regulates effectiveness of ab differentiation of M0b (Hill-type constant)	[0.16,83.97]
k_{m0ad}	Rate of recruitment of M0b by ab	[0.34,181.89]
x_{m0ad}	Regulates effectiveness of recruitment of M0b by ab (Hill-type constant)	[0.01,27.6]
k_{m0p}	Rate of differentiation of M0 by p	$[8.99\times 10^{-3}, 37.2]$
x_{m0p}	Regulates effectiveness of differentiation of M0 by p (Hill-type constant)	$[1.17, 1.14\times 10^{4}]$
k_{m0pb}	Rate of differentiation of M0b by pb	[0.05,89.96]
x_{m0pb}	Regulates effectiveness of differentiation of M0b by pb (Hill-type constant)	$[41.51, 2.92\times 10^{4}]$
k_{m0pd}	Rate of recruitment of M0b by pb	$[4.57\times 10^{-3}, 53.97]$
x_{m0pd}	Regulates effectiveness of recruitment of M0b by pb (Hill-type constant)	[0.24,180.74]
k_{m1p}	Rate of recruitment of M1b by pb	[0.2,92.81]

Table S1 (continued)

Name	Description	Range used
x_{m1p}	Regulates effectiveness of recruitment of M1b by pb (Hill-type constant)	$[9.8\times 10^{-3}, 1.69]$
k_{m2a}	Upregulation of M2b recruitment by a	[0.1,219.93]
x_{m2a}	Regulates effectiveness of M2b recruitment by a (Hill-type constant)	[0.08,94.84]
k_{m2r}	Upregulation of M2b recruitment by R	$[3.61\times 10^{-3}, 20.11]$
x_{m2r}	Regulates effectiveness of M2b recruitment by R (Hill-type constant)	[0.01,18.70]
k_{man}	Rate of M1 switch to M2 by AN	[0.01,27.08]
k_{mne}	Rate of collateral damage to epithelial cells by macrophages and neutrophils	$[1.12\times 10^{-3}, 5.17]$
x_{mne}	Regulates effectiveness of macrophages and neutrophils to damage epithelial cells (Hill-type constant)	[0.03,41.06]
k_n	Rate of migration of Na to lung	$[2.39\times 10^{-3}, 3.54]$
k_{n0p}	Rate of activation of Na by p	[0.01,5.58]
x_{n0p}	Regulates effectiveness of activation of Na by p (Hill-type constant)	[0.03,142.56]
k_{pe}	Production rate of p by Ed	$[44.02, 1.12\times 10^{4}]$
k_{pm1}	Production rate of p by M1 & M1b	[0.24,412.22]
k_{pn}	Production rate of p and pb by neutrophils	$[1.67\times 10^{-3}, 2.95]$
k_{rm2}	Production rate of R by M2	[0.02,40.97]
D_a	Decay rate of a	$[5.16\times 10^{-4}, 5.08]$
D_{ab}	Decay rate of ab	[0.04,12.86]
D_p	Decay rate of p	$[2.76\times 10^{-3}, 41.04]$
D_{pb}	Decay rate of pb	$[4.79\times 10^{-4}, 3.71]$
D_{m0}	Decay rate of M0	[0.01,42.67]
D_{m0b}	Decay rate of M0b	$[7.66\times 10^{-3}, 329.59]$
D_{m1}	Decay rate of M1	$[8.2\times 10^{-3}, 10.16]$
D_{m1b}	Decay rate of M1b	[0.03,60.32]
D_{m2}	Decay rate of M2	[0.27,135.37]
D_{m2b}	Decay rate of M2b	[0.02,16.51]
D_{nb}	Decay rate of Na	$[2.49\times 10^{-3}, 6.03]$
D_{n0b}	Decay rate of N0b	$[3.94\times 10^{-6}, 2.1\times 10^{-3}]$
D_n	Decay rate of N	$[8\times 10^{-3}, 4.32]$
D_R	Decay rate of R	[0.72,761.75]
s_a	Source rate of background ab	$[5.75\times 10^{-3}, 1.11]$
s_m	Source rate of M0b	$[1.28, 1.14\times 10^{-3}]$
s_n	Source rate of N0b	[0.22,225.45]
s_p	Source rate of background pb	$[6.5\times 10^{-4}, 9.4]$

S3. Model Validation

To substantiate the efficacy of the LIRS subsystem, we conducted an open-loop validation process, focusing on the examination of how variations in key parameters impact the evolution of eh. Utilizing a carefully curated subset of the mice experimental dataset, we randomly select 200 representative samples and identify the value ranges for pivotal parameters. By resampling within these ranges while maintaining other parameters constant, we reconstruct an augmented sample set, generating 10 additional cases per original sample, totaling 2000 simulations for every parameter. Each of these simulations is subjected to a standardized ventilation-induced damage scenario ($sd = 0.75$) to evaluate the response of the eh in LIRS model. By comparing the observed trends and phenomena with those reported, the accuracy of the LIRS is verified.

As depicted in Figure S1, our simulations reveal a predictable responsiveness of eh to parameter adjustments, consistent with Minucci's findings and fundamental physiological principles. Specifically, augmenting the baseline repair rate of damaged cells (b_r) leads to a consistent rise in eh. Conversely, elevating the rate of collateral damage inflicted by macrophages and neutrophils on epithelial cells (k_{mne}) results in a decline in eh, particularly acute during the initial 2 hours post-ventilation. Furthermore, bolstering the regulatory capacity of these immune cells to mitigate epithelial damage (x_{mne}) elevates eh, particularly during extended repair phases.

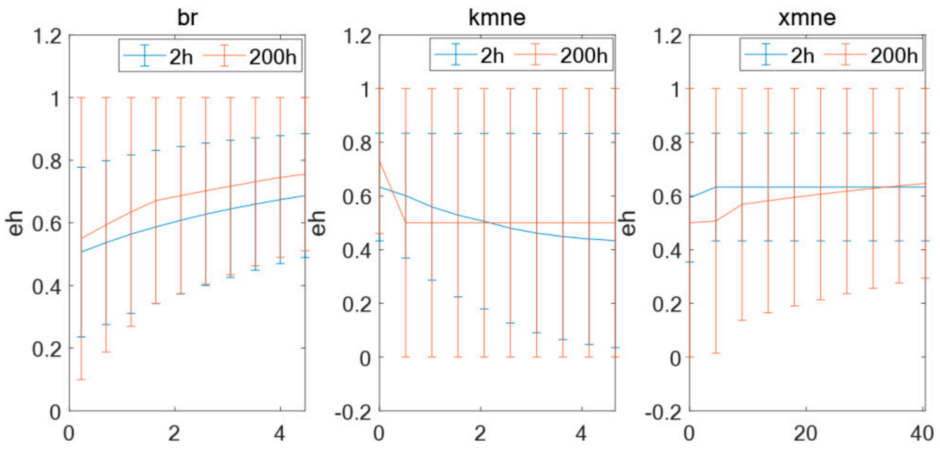

Figure S1. Parameter changes will cause eh changes. With the increase of b_r (baseline repair of damaged cells), the value of eh correspondingly augments. With the increase of k_{mne} (rate of collateral damage to epithelial cells by macrophages and neutrophils), this leads to a decline in eh. With the increase of x_{mne} (regulations effectiveness of macrophages and neutrophils to damage epithelial cells), results in an increase in eh.

Furthermore, to further strengthen the credibility of the TVDS subsystem, we delved into the fundamental workings of the monolayer architecture. This analysis involved utilizing a resistance (R) to monitor the integrity of intercellular tight junctions, a methodology rooted in a paper. As elucidated in Eq. (38), each cell exhibits a maximum resistance of $r = R_{max}N$ when $n = n_{max}$, and a minimum resistance of $r = R_{min}N$ when $n = 0$. Drawing upon the empirical data from Bates' studies, we assign $R_{max} = 6 \times 10^6\Omega$, $R_{min} = 1 \times 10^6\Omega$, and $\gamma = 0.2$. By calculating the R_{mon} of the cell layer from the individual cell resistances (r), we are able to track its dynamic behavior. This approach allowed us to not only track temporal changes in tight junction integrity but also validate our model's efficacy by comparing the observed R_{mon} behavior with literature report.

$$r = \begin{cases} R_{max}N \ , if\ n_{broken} \le \gamma n_{max} \\ R_{min}N + N(R_{max} - R_{min})\left(\frac{n}{(1-\gamma)n_{max}}\right) \ , if\ n_{broken} > \gamma n_{max} \\ R_{min}N \ , if\ n_{broken} = 25\ or\ n = 0 \end{cases} \quad (38)$$

As for the selection of parameters in TVDS model. Based on the fact that the weight of the mouse used in the physiological experiment is 22 grams, the value of A is determined to be 4.5ml/kg after numerical conversion. However, the principle of lung protective ventilation mentioned in the existing research advocates that the tidal volume should be set in the range of 4ml/kg to 8 ml/kg during mechanical ventilation. Therefore, based on the principle of lung protective ventilation, the critical tidal volume V_{tcrit} is set to 4 ml/kg. And each RD cycle lasting 20 seconds occurring 180 times within an hour.

We present in Figure S2 the outcomes of an open-loop validation study of the TVDS, demonstrating biphasic epithelial dysfunction processes under varying tidal volumes. As shown in Figure S2, these phases—a stationary state (Phase 1) followed by an exponential decay (Phase 2)—align with animal experimental observations. With increasing tidal volumes, Phase 1 diminishes sooner, expediting the exponential deterioration in Phase 2, highlighting the TVDS system's sensitivity to tidal volume modulations and their crucial implications for lung health.

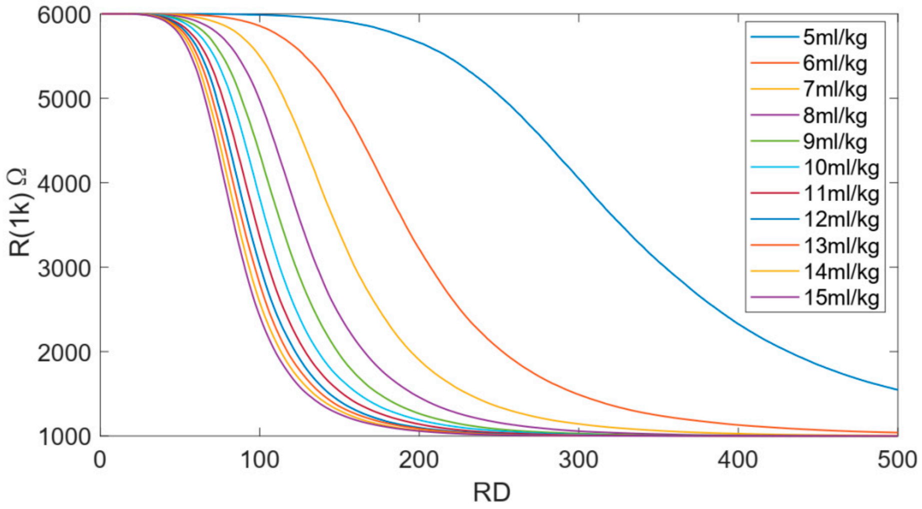

Figure S2. Different evolution processes of different tidal volumes. The variation curve of R (R_{mon}) within recruitment/derecruitment (RD) process in the TVDS, when subjected to varying tidal volumes, exhibits distinct patterns. Curves of different colors represent different tidal volumes. The curves display two distinct phases: Phase 1, characterized by a stationary state, and Phase 2, marked by an exponential decay state.
